# A Novel Water-Shore-Line Detection Method for USV Autonomous Navigation

**DOI:** 10.3390/s20061682

**Published:** 2020-03-18

**Authors:** Xiong Zou, Changshi Xiao, Wenqiang Zhan, Chunhui Zhou, Supu Xiu, Haiwen Yuan

**Affiliations:** 1 School of Navigation, Wuhan University of Technology, Wuhan 430063, China; zx2000@whut.edu.cn (X.Z.); zwq626197298@whut.edu.cn (W.Z.); sp_xiu@whut.edu.cn (S.X.); hw_yuan@whut.edu.cn (H.Y.); 2 Hubei Key Laboratory of Inland Shipping Technology, Wuhan 430063, China; 3 National Engineering Research Center for Water Transport Safety, Wuhan 430063, China; 4 Intelligent Transportation Systems Research Center, Wuhan 430063, China

**Keywords:** USV (unmanned surface vehicle), WSL (water-shore-line), LSD (line segment detector), epipolar constraint

## Abstract

For the navigation of an unmanned surface vehicle (USV), detection and recognition of the water-shore-line (WSL) is an important part of its intellectualization. Current research on this issue mainly focuses on the straight WSL obtained by straight line fitting. However, the WSL in the image acquired by boat-borne vision is not always in a straight line, especially in an inland river waterway. In this paper, a novel three-step approach for WSL detection is therefore proposed to solve this problem through the information of an image sequence. Firstly, the initial line segment pool is built by the line segment detector (LSD) algorithm. Then, the coarse-to-fine strategy is used to obtain the onshore line segment pool, including the rough selection of water area instability and the fine selection of the epipolar constraint between image frames, both of which are demonstrated in detail in the text. Finally, the complete shore area is generated by an onshore line segment pool of multi-frame images, and the lower boundary of the area is the desired WSL. In order to verify the accuracy and robustness of the proposed method, field experiments were carried out in the inland river scene. Compared with other detection algorithms based on image processing, the results demonstrate that this method is more adaptable, and can detect not only the straight WSL, but also the curved WSL.

## 1. Introduction

With the development of the economy and water transportation, the importance of the inland waterway is increasingly prominent. The maritime supervision system relies mainly on the vessel traffic services (VTS), automatic identification system (AIS), maritime digital close circle television (CCTV) and maritime patrol crafts [1], etc. However, they have lagged far behind with the needs for “smart maritime”.

Particularly for the development of "e-navigation" technology, unmanned surface vehicle (USV) is widely used to perform hazardous and time-consuming missions, such as regular inspections, maritime monitoring, disaster emergency, search and rescue, etc., due to its low risk and high mobility characteristics [2]. In reality, the intellectualization of USV is still far from actually being “unmanned”. 

To achieve autonomous cruise and complete various tasks, the USV needs to be equipped with different sensors, such as GPS, inertial measurement unit, radar, camera, etc., to sense and understand the surrounding environment, including autonomous target detection, recognition and tracking. Coupled with the development of computer vision technology, the camera has become an indispensable sensor device for USV because of its low cost, rich information and high resolution [3].

The water-shore-line (WSL) is one of the most prominent symbols of optical images captured by boat-borne vision in an inland river scene. First of all, due to the height limitation of USV, obstacles in the field of vision always appear near WSL, so accurate WSL detection is very beneficial for target detection and recognition. Secondly, WSL detection can not only obtain the motion state and surrounding environment information of the USV, but also estimate the location information of the USV from the shore [4].

WSL detection is similar to sea-sky-line [4,5,6,7], water-sky-line [8,9,10] and skyline detection [11], which is a connection line with a larger gradient value in the image. Compared with WSL detection, there are many studies on sea-sky-line detection, mainly including image processing methods such as straight line fitting [4,5,6,7], wavelet transform [12], region growth [13], threshold segmentation [9,14] and other methods [15].

An Bowen et al. [4] first applied the OTSU algorithm to complete image binarization, then extracted the line segments with the Hough transform, and finally used the line segment fitting to obtain the sea-sky-line. Liang et al. [6] used texture features to narrow the sea-sky-line area, then got candidate points in the area and applied the improved line segment fitting method to obtain the line segment parameters. Ma et al. [7] analyzed the adverse effects of waves, clouds and glaring reflection light on sea-sky-line detection in the image, and proposed using the line segment detection to get candidate points, and then applied the least squares method to fit the sea-sky-line. It has a certain inhibitory effect on noises such as water ripples.

The literature [11] compared five common methods of sea-sky-line detection, and analyzed the improvement measures of these methods. From the experimental comparison, it can be seen that the combination algorithm of Canny and Hough performs well with regards to accuracy and time consumption.

In addition to the method of straight line fitting, Yang et al. [12] proposed a multi-level wavelet transform to suppress clutter noise in the image, and a multi-directional Gabor filter to enhance the sea-sky-line edge. Wang et al. [13] proposed a method that first obtained a gradient saliency image, then used the region growth method to generate a support region, and combined the spatial characteristics to realize sea-sky-line detection. Chiyoon et al. [15] proposed a sea-sky-line detection method which combined the edge information of different scales and used CNN to validate edge pixels belonging to the horizon, and then the sea-sky-line was estimated iteratively by linear curve fitting. 

The WSL and the sea-sky-line are similar; both of them are composed of larger gradient values in the image, but there are also many differences. Because of the huge scene, the imaging of the sea-sky-line is relatively blurred, and related research often enhances the blurred target from the image. However, for the WSL image of the inland river, it is obvious that the scene is not as large; the image is clearer (except for the fog), but the environment is more complicated, with high buildings, trees, grass and bridges in the background, as well as natural disturbances of rain, snow, fog and wind, especially the water ripple caused by strong winds, similar to WSL. All these have brought adverse effects to the detection and recognition of WSL. Therefore, the studies on WSL detection are mainly carried out to delete the interference target in the image.

In the field of WSL detection, there are also many recent research results. Dong et al. [16] proposed a method similar to sea-sky-line detection, which included image downsampling, image denoising, edge detection, line segment extraction and straight line fitting.

In response to irregular texture information by wind and rain in the image, Wei et al. [8] proposed a method based on structure extraction and texture analysis, which used the gray level co-occurrence matrix to calculate the texture information of the image, and then introduced the structure extraction technology to remove the texture information in the image. Subsequently, combined with optical shadow processing and energy optimization, they proposed an automatic water boundary detection method, which performed background segmentation on shadow-insensitive intrinsic images, and transformed the water boundary segmentation problem into differential energy equations, and adopted iterative optimization to find the optimal solution [9]. 

In view of the background texture and water surface reflection interference in WSL images, our team has also done relevant research. Zhan et al. [10] applied a local variational method to smooth the images, and then used the RANSAC algorithm to constrain these waterline points near a straight line. Subsequently, the paper [17] proposed a visual detection method to perceive navigable water via the online training of neural networks. Firstly, different regions of the image were clustered, and labels and confidence values were allocated, and then they were fed into the training grid of neural networks. Finally, the training grid was utilized to segment the input image again, but with higher precision and robustness.

In the above algorithms, the straight line fitting method obviously cannot detect the curved WSL, and the machine learning method is too computationally intensive. On the one hand, online learning cannot achieve a real-time effect. On the other hand, offline learning will result in a great discount in detection accuracy due to the variety of scenes.

Aiming at this problem, a novel three-step approach is proposed for various WSL detections in this paper. Firstly, the line segment pool is built by the line segment detector (LSD) [18] algorithm. Then, the line segment pool is roughly screened by the instability of the water area, and the stable line segment pool is refined by the epipolar constraint to obtain accurate and a stable onshore line segment pool. Finally, the complete shore area is obtained through the onshore line segment pool of the multi-frame images, whose lower boundary is the desired WSL. This method can detect not only the straight WSL, but also the curved WSL.

The remainder of this paper is organized as follows: the framework and process of the proposed method is introduced in Section 2, including line segment detection, construction of the onshore line segment pool and generation of the WSL. Section 3 describes the experimental platform, equipment and sites. Section 4 is the experimental section, which verifies the validity of the method through field experiments and comparisons with other methods. Finally, Section 5 gives some conclusions.

## 2. The Proposed Method

The onboard vision is placed upright on USV’s bow, and it has a wide field of view without obstruction. In the face of inland water, the acquired images include the sky, bank and water surface. The boundary between bank and water surface is called water-shore-line, which is the connection of the larger gray gradient of pixels. For WSL detection, many researchers adopt the line segment fitting methods [10,16], which assume the WSL to be a straight line, but these methods significantly limit their applications.

This paper finds a novel way to separate the shore area from the water area, which explores their intrinsic attributes and distinguishes whether an object belongs to the shore area or the water area. The method can detect both the straight WSL and the curved WSL.

### 2.1. Framework

**Figure 1:**
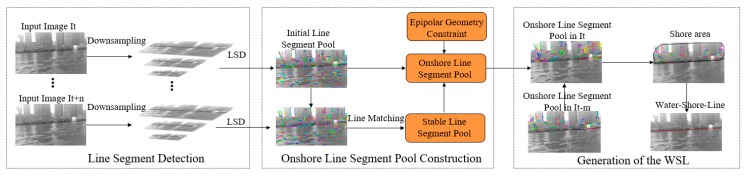
The framework of the water-shore-line (WSL) detection method for unmanned surface vehicle (USV) in inland river scene.

The proposed method is designed as a framework, shown in Figure 1 with three steps: line segment detection, construction of the onshore line segment pool, and generation of the WSL. Line segment detection is based on the LSD algorithm, through which the initial line segment pool can be obtained, which includes not only the line segment generated by the onshore objects (tall buildings, trees, etc.), but also the line segment generated by the water ripple. In order to improve the operation’s efficiency, the high-resolution image is downsampled before the line segment detection.

The construction of the onshore line segment pool is divided into two steps: firstly, using the extremely unstable characteristics of the water surface, most of the water surface line segments are removed to obtain a stable line segment pool; then, this pool is purified by the epipolar constraint between image frames to obtain an accurate and stable onshore line segment pool. 

The shore area is obtained by combining the onshore line segment pool of multi-frame images, and its lower boundary is the desired WSL.

### 2.2. Line Segment Detection

Hough transform is a well-known line segment detection algorithm, but its time complexity is$\;O\left(N^{2}\right)$, which leads to high time cost. In contrast, the time complexity of the LSD algorithm is O(N), and it can quickly detect line segments in the image, and so the LSD algorithm is adopted in this paper.

The LSD algorithm first calculates the gradient size and direction of the pixel points in the image, and takes the points with small gradient direction change and which are adjacent to each other as a connected domain, and then judges whether it needs to be disconnected to form a larger rectangular domains according to the rectangularity of each domain. Finally, all the generated domains are improved and filtered, retaining the field which meets the conditions, that is, the final line detection results. The specific steps are as follows: (1) constructing the state list: calculate the gradient value and gradient angle of each pixel in the image, and perform pseudo-sorting according to the gradient value. (2) Rectangular fitting: select the largest gradient as the seed point, and diffuse the region according to the direction of the gradient angle. (3) Verification line segment: calculate the probability that the number of interior points in the rectangular region is less than that in the same region in the perfect noise image (the number of false alarms, NFA).

**Figure 2:**
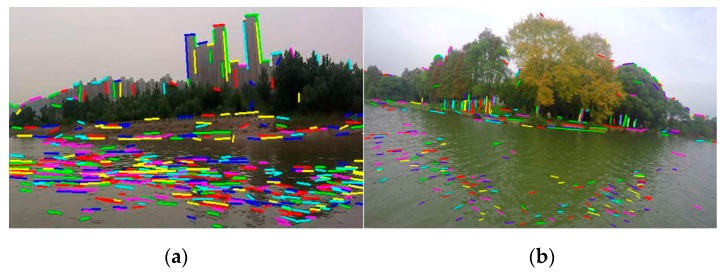
The results of line segment detection by the line segment detector (LSD) algorithm: (**a**) the line segments in the image include water ripple, trees, buildings, fences, shorelines, etc.; (**b**) the shoreline in the image is not straight.

**Figure 3:**
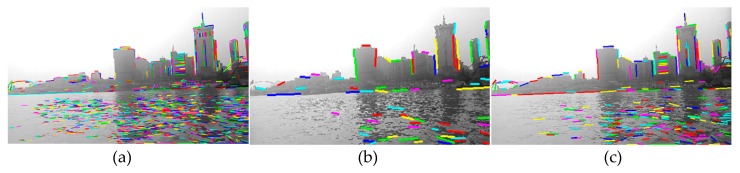
The relationship between the image resolution and the number of line segments: (**a**) the image resolution is 1280 × 720 px, and a large number of line segments are detected; (**b**) the image resolution is 320 × 180 px, and many line segments are lost in the image; (**c**) the image resolution is 640 × 360 px, and the number of line segments in the image is more appropriate.

The LSD algorithm can detect a large number of line segments in the image, but the algorithm alone is not suitable for our WSL detection. The reasons are as follows:

(1) The LSD algorithm can detect line segments, but it has no distinguishing mechanism and cannot distinguish which line segments belong to the shoreline. Figure 2a shows the results of line segment detection by the LSD algorithm, including line segments generated by water ripple and onshore buildings. In order to extract the WSL, it is necessary to remove the line segments belonging to the water surface and buildings.

(2) Even if the LSD algorithm can detect a straight shoreline, it is powerless in the case where the shoreline is not straight, as shown in Figure 2b, where it is obvious that the shoreline is curved. In fact, the shoreline in Figure 2a is not a straight line.

(3) The higher the image resolution, the more line segments will be extracted, and the corresponding operation will be more time-consuming. However, if the resolution is too low, many line segments will be lost, which will lead to many missed detections. As shown in Figure 3, a large number of line segments can be detected from Figure 3a (1280 × 720 px), while the number of line segments detected in Figure 3b (320 × 180 px) is significantly reduced, and many line segments that could be detected in high-resolution images are ignored. For the shoreline image, in order to find the appropriate image resolution and the number of line segments, it is necessary to select the appropriate sampling scale. In this paper, the method of reference [19] is used to select the scale adaptively: find out and make the energy function *E* minimum, which is as follows:

(1) $$E\left(\sigma \left(x,y\right)\right)=\iint^{\;}\left[{\left(f\left(x,y\right)-G\left(\sigma \left(x,y\right),x,y\right)\;*f\left(x,y\right)\right)}^{2}+\lambda {\left|\nabla \frac{1}{\sigma \left(x,y\right)}\right|}^{2}\right]dxdy,$$

where f(x,y) represents image, G(σ(x,y),x,y) represents gaussian filter, σ(x,y) represents the scale size of gaussian filter, ∇ represents partial derivative and λ is constant. In the original text, the optimal filtering scale is obtained by the initial value plus iteration. However, this method is complicated and computationally intensive. In this paper, the resolutions, such as 1280 × 720 px, 640 × 360 px and 320 × 180 px, are selected directly, and the corresponding energy functions are calculated respectively, and then the image with the lowest energy value is selected. As shown in Figure 3, the resolution 640 × 360 px is selected because the energy value of Figure 3c (640 × 360 px) is lower than that of Figure 3a,b.

### 2.3. The Construction of the Onshore Line Segment Pool

Through the above steps, the initial line segment pool is obtained, but it includes all the line segments in the image, which are generated by both onshore objects and the water ripple. Here, the coarse-to-fine strategy is adopted to obtain the onshore line segment pool from the initial line segment pool. Firstly, the unstable line segments in the initial line segment pool are removed by line segment matching in the image sequence. Most of the reserved line segments belong to onshore line segments, because the line segments in the water area are very unstable, which is demonstrated in detail in this article. Then, the epipolar constraint between the image frames is used to purify the above line segments to obtain an accurate and stable onshore line segment pool. The details are described in the following subsections.

#### 2.3.1. Rough Selection of Onshore Line Segment Pool

As we all know, buildings are static in the real world, and the water surface will undulate up and down for various reasons, resulting in a variety of water ripples. What about the shoreline image acquired by the visual system? In order to compare the differences of line feature between the water area and non-water area in the image, the survival time of line features in the image sequence was calculated statistically.

It was found that the line features of water area are extremely unstable, which usually disappear after the next frame or several frames, while the line features in the non-water area were relatively stable, and even that some line features always exist until the scene leaves the camera’s field of view. In fact, the instability of line features in water area is caused by the influence of the wind, waves and current, which results in the short lifetime of line features. In addition, since the water surface is a non-Lambert surface, this will also exacerbate the change of line segment imaging. The following is a detailed description.

**Figure 4:**
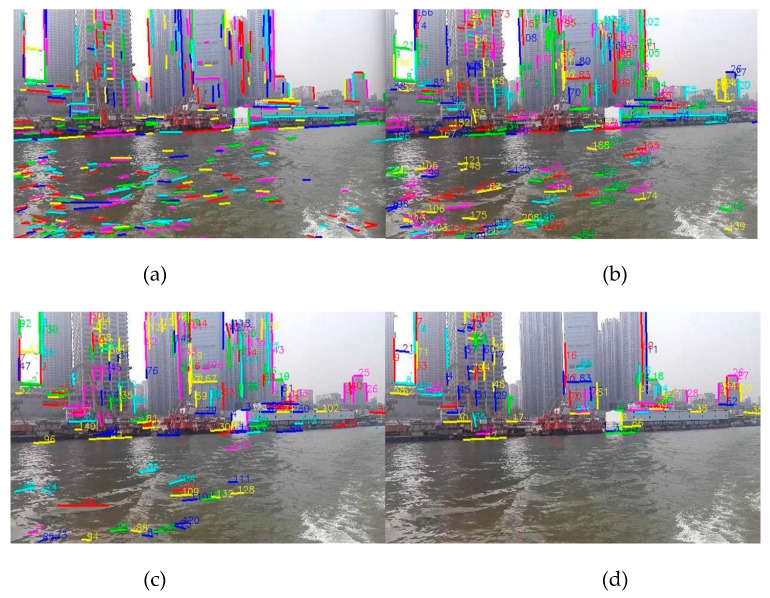
The results of line segment extraction and matching: (**a**) line segment extraction of the first frame image; (**b**) the line segment matching result of the first frame and the second frame image; (**c**) the result of line segment matching with the third frame on the basis of Figure 4b; (**d**) the result of line segment matching that repeats until the 10th frame.

In order to compare the performance of line features between the water and non-water area, the matching experiments of line features in shoreline images had been carried out. As shown in Figure 4, Figure 4a (640 × 360 px) is the result of extracting the line segment features in the image by the LSD algorithm. Figure 4b is the result of matching the first frame with the second frame of the image sequence using the line segment matching algorithm [20]; the number indicates the serial number of the matched line segment. Figure 4c is the result of matching with the line features of the third frame on the basis of Figure 4b. Additionally, Figure 4d is the result of line segment matching that repeats until the 10th frame. It can be clearly seen from the figures that the line features of the water area are reduced more rapidly than those of the shore area. In other words, the line features in the water area are unstable, and at very short intervals, the matched line features in the water area are gradually reduced to none, but the line features in the non-water area still maintain a stable match.

**Figure 5:**
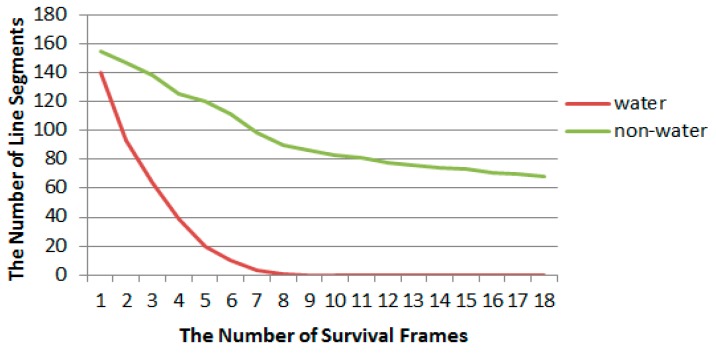
Survival frames statistics of line segments in water and non-water area; the red and green respectively correspond to the water and non-water area.

In order to more clearly compare line features and the differences between water and non-water area, on the basis of above line segment matching, the survival time of the line features is statistically analyzed for the image sequence. As shown in Figure 5, the horizontal axis represents the lifetime of line features through the number of the image frame, and the vertical axis represents the number of line features matched; the red and green respectively correspond to the water and non-water area in the image. It can be seen from the figure that the lifetime of line features in the water area is very short, that is to say, the experiment demonstrates that the line features in the water area are more unstable than in the non-water area.

In order to further verify this characteristic, and to confirm which factors are related to the instability of line features in the water area, the tracking experiment of line features has been further improved: under different weather conditions, 10 sets of image sequences are respectively selected from Site 1 and Site 2 (in Section 3), each of which contains 100 images. The line features of the initial image are extracted, which are matched and tracked for subsequent image frames, and the average survival time of the water area and non-water area line features is counted.

**Figure 6:**
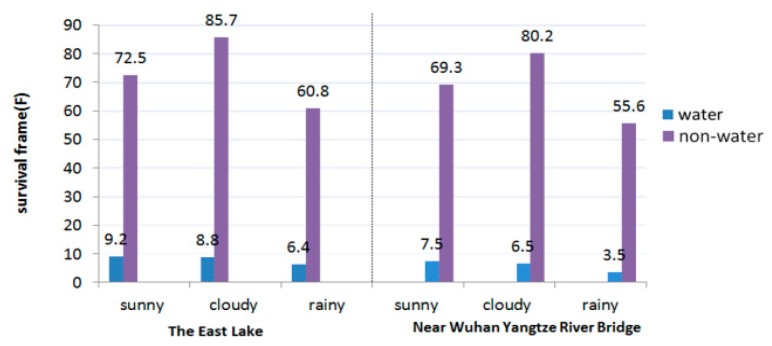
Average survival frames of line segments on the East Lake and near Wuhan Yangtze River Bridge; the blue and purple respectively correspond to the water and non-water area.

As shown in Figure 6, the left and right correspond to the results of Site 1 and Site 2, respectively. The three columns of data correspond to sunny, cloudy and rainy days; blue represents the water area, and purple represents the non-water area. The data show that the average survival time of the water area and non-water area line features differs by an order of magnitude, which fully demonstrates that the line features in the water area are unstable, especially in the case of rainfall, which will lead to the rapid change of water ripple, and the instability is likely to be caused by wind, wave and current in the environment.

#### 2.3.2. Fine Selection of Onshore Line Segment Pool

Usually, the stable line segment pool is obtained by utilizing the above-mentioned water area instability characteristics, and it can represent the onshore line segment pool well. As shown in Figure 4d, all the line segments are onshore line segments. However, due to the disturbance in the scene and mismatching, if the line segments of the water area are mixed into the onshore line segment pool, this will have an adverse effect on subsequent operation. For this, the epipolar constraint is introduced to further select the above line pool, so as to obtain the accurate and stable onshore line segment pool.

The corresponding feature points in sequence images are constrained by epipolar geometry, which is usually used to accelerate the matching process of feature points. The fundamental matrix is an algebraic expression of epipolar geometry:

(2) $$\;P^{\prime }{}^{T}FP=\left(\begin{array}{ccc} u_{\;}^{\prime } & v_{\;}^{\prime } & 1 \end{array}\right)\left(\begin{array}{ccc} f_{1} & f_{2} & f_{3}\\ f_{4} & f_{5} & f_{6}\\ f_{7} & f_{8} & f_{9} \end{array}\right)\left(\begin{array}{c} u\\ v\\ 1 \end{array}\right)=0,$$

where F is the fundamental matrix, $P={\left(\begin{array}{ccc} u & v & 1 \end{array}\right)}^{T}$ and$\;P^{\prime }={\left(\begin{array}{ccc} u_{\;}^{\prime } & v_{\;}^{\prime } & 1 \end{array}\right)}^{T}$ are the homogeneous coordinates of the corresponding feature points in the images. If it is represented by epipolar line,

(3) $$L_{e}{}^{T}P\prime =0,$$

which represents P′ on the epipolar line${\;L}_{e}$, where epipolar line

(4) $$L_{e}=F^{T}P^{\prime }={\left(\begin{array}{ccc} a_{e} & b_{e} & c_{e} \end{array}\right)}^{T},$$

where $a_{e}$ and $b_{e}$ are the coefficients of the line, $c_{e}$ is a constant. Similarly, P is also on the epipolar line$\;L_{e}$. Next, epipolar geometry is used to refine the above matching line segments. 

The first step is to find the fundamental matrix F. When the matching points between images exceeds eight pairs, considering that there may be outliers, the RANSAC method is generally adopted to deal with the problem: eight pairs of matching points are randomly selected to solve the fundamental matrix F, and all matching points are used to calculate the error. If the error of a pair of matching points is lower than the threshold, it is an inlier, otherwise it is an outlier. If continuous iteration until the maximum number of iterations or the proportion of inliers is greater than the given threshold, then the F of the maximum number of inliers is taken as the initial solution. Finally, all inliers are used for the non-linear optimization of F [21].

After the fundamental matrix is obtained, the distance between two line segments is defined as the square of the distance between the midpoint of the line segment and the corresponding epipolar line, which are used to measure the matching degree of the two line segments. Specifically, if the line segment${\;L}_{1}$ and${\;L}_{2}$ are matching lines, then the midpoint${\;X}_{1}\;$of $L_{1}$ and the midpoint $X_{2}\;$of $L_{2}$ are matching points, where the corresponding matching points can be selected by the distance criterion [22], that is, the Euclidean distance from the matching point to the corresponding epipolar line. Its function is expressed as follows:

(5) $$d\left(L_{i},{L^{\prime }}_{i}\right)=d^{2}\left(P_{i},L_{e}\right)+d^{2}\left({P^{\prime }}_{i},{L^{\prime }}_{e}\right)=\frac{{\left({P^{\prime }}_{i}{}^{T}FP_{i}\right)}^{2}}{{\left(FP_{i}\right)}_{1}{}^{2}+{\left(FP_{i}\right)}_{2}{}^{2}}+\frac{{\left({P^{\prime }}_{i}{}^{T}FP_{i}\right)}^{2}}{{\left(F^{T}{P^{\prime }}_{i}\right)}_{1}{}^{2}+{\left(F^{T}{P^{\prime }}_{i}\right)}_{2}{}^{2}}\;,$$

where${\;L}_{i}$ and${\;L\;\prime }_{i}$ represent the i-th pair candidate matching line in the two images, $P_{i}$ and ${P^{\prime }}_{i}$ are respectively their midpoints,$\;L_{e}^{\prime }=FP_{i}$ represents the epipolar line corresponding to point$\;P_{i}$,$\;d\left({P^{\prime }}_{i},{L^{\prime }}_{e}\right)$ represents the euclidean distance from point$\;P^{\prime }{}_{i}$ to the epipolar line$\;L_{e}^{\prime }$, that is, the distance from point x to line y is calculated by Equation (6).

(6) $$d\left(x,y\right)=\frac{y^{T}x}{\sqrt{y_{1}{}^{2}+y_{2}{}^{2}}}\;,$$

where $y_{i}$ is the vector form of line y.

**Figure 7:**
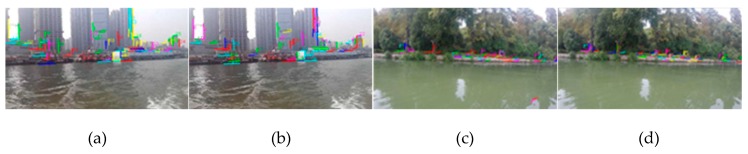
The results of line segment pool fine selection: (**a**) the result of line segment pool rough selection (that is Figure 4d); (**b**) the result of line segment pool fine selection on the basis of Figure 7a; (**c**) the result of line segment pool rough selection in the Yangtze River; (**d**) the result of line segment pool fine selection on the basis of Figure 7c.

In order to compare the effect of the fine selection of the onshore line segment pool, the fine selection experiment was carried out. As shown in Figure 7, Figure 7a is the result of line segment pool rough selection (that is Figure 4d), Figure 7b is the result of line segment pool fine selection on the basis of Figure 7a. Similarly, Figure 7c is the result of line segment pool rough selection in the Yangtze River and Figure 7d is the result of line segment pool fine selection on the basis of Figure 7c.

It can be seen from the figure that, for the calmer water surface of Figure 7c, water surface line segments can be removed well, while the onshore line segments remain stable. On the other hand, for the water area with great fluctuation (Figure 7a), because the rough selection has already removed the water surface interference, the fine selection results have not changed much.

**Figure 8:**
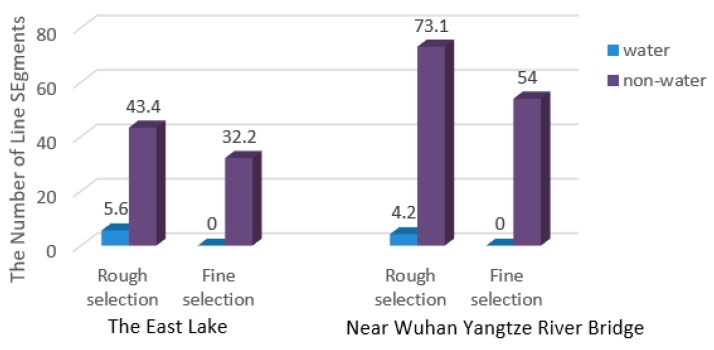
Average number of line segments on the East Lake and near Wuhan Yangtze River Bridge; the blue and purple respectively correspond to the water and non-water area.

In order to further evaluate the effect of the fine selection of onshore line segment pool, 10 sets of image sequences were respectively selected from Site 1 and Site 2 at random. The number of line segments in non-water and water area before and after fine selection was counted. As shown in Figure 8, the left and right correspond to the results of Site 1 and Site 2 respectively, blue represents the water area, and purple represents the non-water area. 

It can be seen from the figure that, whether it is the East Lake or the Yangtze River, line segments in the water area are reduced to zero, and line segments in the non-water area are slightly reduced, which indicates that the fine selection can remove line segments in the water area well.

### 2.4. Generation of the WSL

Through the above processes, the onshore line segment pool of a certain frame image (such as ti time) is obtained. The following describes how to generate the shore area by the pool and make this area more complete using multi-frame image information, and the lower boundary of the area is the desired WSL.

#### 2.4.1. Generation of the Shore Area

For a frame of the image sequence, the onshore line segment pool corresponding to the frame is obtained by the coarse-to-fine strategy above-mentioned. Next, the shore area is generated by these line segments, that is, the largest polygon area is generated by the endpoints of all lines. The common method is to randomly select several points to form a polygonal area, and then use the ray-casting algorithm and its improved algorithm [23,24] to determine whether the next point is in the area, and if so, the point is deleted, otherwise, the point is connected to the nearest vertex of the polygon to form a larger area, until all the points are operated to generate the largest polygon area.

**Figure 9:**
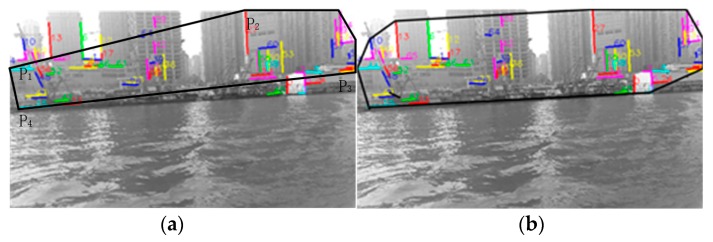
The shore area is generated by the single image; (**a**) effective selection of initial values to generate polygon area, (**b**) acquisition of the shore area.

The complexity of the ray-casting algorithm is O(p×q), where p and q are the number of points and polygon boundaries, respectively. Due to a large number of points, this paper attempts to use a simpler strategy through the special spatial position characteristics of the polygon area: 1) according to the coordinates of the endpoints of a line segment, the four points ($P_{1}$, $P_{2}$, $P_{3}$, $P_{4}$) with the maximum and minimum coordinates are found and connected to form a polygon (Figure 9). 2) when judging whether a point is in a polygon or not, it is not necessary to operate on all points, and only necessary to select points whose ordinates are less than $P_{1}$ or $P_{2}$ and points whose ordinates are larger than $P_{3}$ or $P_{4}$. This can not only reduce the adverse effects of random initial values, but also greatly cut down the number of operating points.

Using the time period from $t_{i}$ to${\;t}_{i+n}$, the onshore line segment pool at $t_{i}$ time is obtained by the above method, and then the shore area is generated. As shown in Figure 9b, it can be seen that the area is not perfect enough. The main reason is that only the information from $t_{i}\;$time to $t_{i+n}\;$time is used for the current image, but the information before${\;t}_{i}\;$time, such as the information from $t_{i-m}$ time to $t_{i}$ time, is not used. 

**Figure 10:**
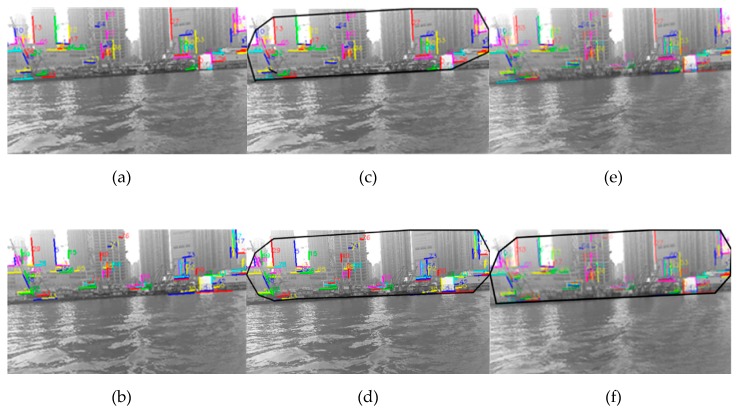
The shore area is generated by the image sequence: (**a**) the onshore line segment pool at $t_{i}$ time using $t_{i}$ time to $t_{i+n}$ time, (**b**) the onshore line segment pool at $t_{i-m}$ time using $t_{i-m}$ time to $t_{i}$ time, (**c**) the shore area corresponding to Figure 10a, (**d**) the shore area corresponding to Figure 10b, (**e**) the onshore line segment pool at $T_{i}$ time combined with Figure 10a,b; (**f**) the shore area corresponding to Figure 10e.

In response to this problem, in order to obtain a relatively perfect shore area at${\;t}_{i}$ time, we used not only the information after $t_{i}$ time but also before${\;t}_{i\;}$time, that is, the time from $t_{i}$ to $t_{i+n}\;$and the time from $t_{i-m}$ to${\;t}_{i}$. The former corresponds to Figure 10a, the latter corresponds to Figure 10b–d, respectively, correspond to their shore areas. Due to the fact that the time interval between images is very short, for simplicity, without considering the shift of points and line segments, the onshore line segment pool combining the two time periods is obtained as shown in Figure 10e, and it is defined as the onshore line segment pool at${\;T}_{i}$ time. Finally, the shore area in the image is obtained by the above method, as shown in Figure 10f. It can be seen that the Figure 10f is better than the Figure 10c,d. In other words, the more valid the information used, the better the result.

#### 2.4.2. Generation of the WSL

Through the above steps, the shore area has been obtained, and then the spatial position characteristics in the image can be utilized: the shore area is above the water area, therefore, the lower boundary of the shore area is the shoreline. Since the purpose of this article is mainly to detect WSL, in order to further reduce the calculation of the shore area generation, it is assumed that the upper left corner and upper right corner of the image are two reference points which belong to the shore area.

## 3. Experimental Equipment and Sites

### 3.1. Experimental Equipment

**Figure 11:**
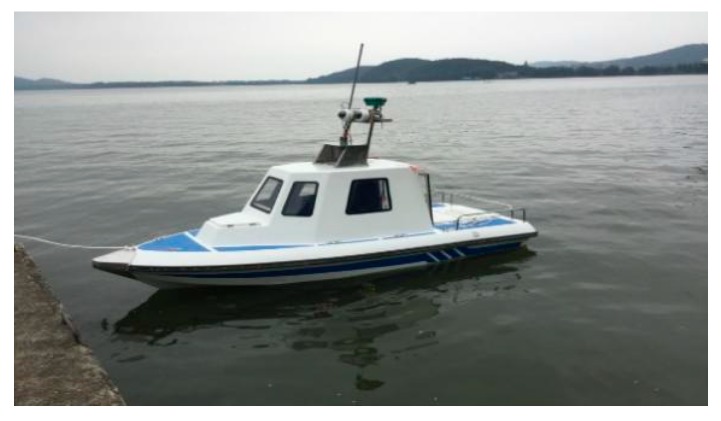
The iNav-II USV.

The iNav-II USV is a 4.15 m-long and 1.6 m-wide vessel, which is our design and development (Figure 11). The USV adopts single pod propeller, and deflection angle of the propeller is limited to −25°~ 25°. The whole ship power and equipment power are supplied by two sets of 48v lithium batteries.

**Figure 12:**
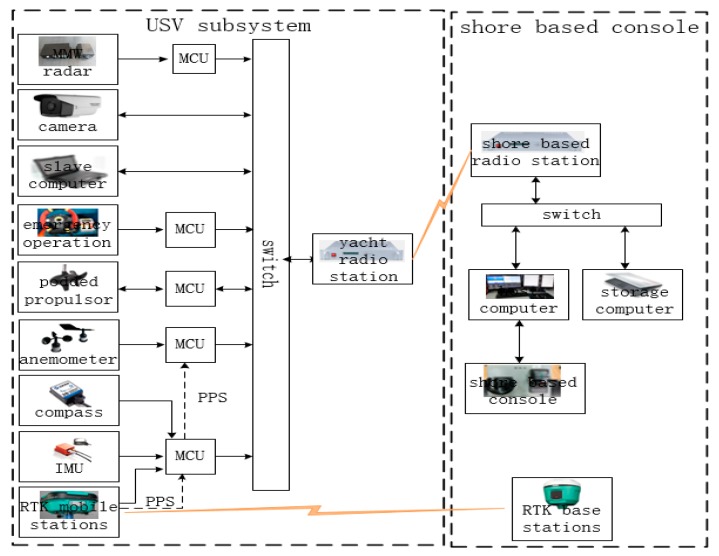
The system architecture of the iNav-II USV.

The USV system consists of two parts: the USV subsystem and the shore-based support system (Figure 12). The USV subsystem mainly includes a differential GPS mobile station, inertial measurement unit, electronic compass, boat-borne radio, wind speed and direction meter, emergency maneuvering device and control computer. The boat system uses pulse per second (PPS) of the differential GPS mobile station to achieve precise timing. The data of each sensor and device in the boat system is packaged by MavLink protocol and then transmitted and communicated by switch and radio station.

The proposed method has been implemented on PC with a double-core Intel i5 processor and 8 GB memory, which is programmed in Visual C++ 2015 using OpenCV SDK 3.0. The vision system consists of an industrial camera (200W pixel, 3.2 um pixel size) and industrial lens (2.8–12 mm, 1:1.4), and the captured images were recorded at 30 f/s, with a resolution of 1280 × 720 pixels. The details of USV and the computer for the experiment are shown in Table 1.

### 3.2. Experimental Sites

Three sites were selected as our experimental bases. Site 1 was the second largest city lake in China, Wuhan East Lake water. The site water was wide and unobstructed. The wind speed on the lake surface was about 0.8~3.5 m/s on the day of the experiment, and the water flow speed in the experimental water was about 0.0–0.3 m/s. Apart from the speed of water flow, others were similar to real scenes of inland water. Sites 2 and 3 were near Wuhan Yangtze River Bridge and the Second Yangtze River Bridge of Wuhan, respectively. Owing to the strict management of the Yangtze River, our USV was prohibited, the image sequences were collected on ferries. The data collection was conducted on May 20, 2017, during cloudy weather conditions, light breeze (wind speeds of 1.6–3.3 m/s), visibility >5 km and water flow velocity 1.0–1.5 m/s,according to data from the Wuhan National Basic Weather Observation Station and the Wuhan Maritime Safety Administration. In order to expand the data set, multiple sets of data were reacquired later under different weather conditions, as shown in Table 2.

## 4. Results and Discussions

In order to verify the effectiveness of the method in inland river scenes, we collected a large number of image sequences in the Yangtze River without a known data set. Moreover, the USV was used in the East Lake for expanding our own data sets, including different lighting, weather, background, and so on.

### 4.1. Experiments

In this section, two groups of experiments are carried out under different conditions. The WSL of the first group is an approximately straight line, and the second group is a non-straight line. The first group was divided into four experiments according to changed conditions. The experimental conditions are shown in Table 3.

**Figure 13:**
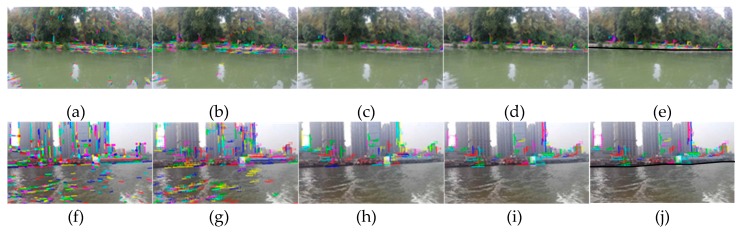
The WSL generation experiments for small scenes: (**a**–**e**) is the experiment I in the East Lake, which is line segment extraction, line segment matching, line segment pool rough selection, line segment pool fine selection and WSL generation. (**f**–**j**) is the experiment II, which is in the Yangtze River.

In experiment I, the USV is very close to the shore and the speed of the USV is very slow in the East Lake. The acquired image is very clear, from which a large number of line segments can be detected, and the image resolution was chosen to be 640 × 360 px through the energy function (3-1). Figure 13a is the result of extracting the line segment, Figure 13b is the result of the line segment matching of the initial two frames of images, Figure 13c shows the stable line segment pool obtained by rough selection (time interval is 10 frames), Figure 13d shows onshore line segment pool by fine selection, and Figure 13e shows the generated WSL (m, n take 10 frames).

As can be seen from the figures, because the USV’s speed is very slow and the water surface is relatively calm, in the tenth frame, there are two water surface line segments remaining in the pool through rough selection, which are then removed through fine selection, and finally the WSL is obtained by the formed shore area.

Experiment II is in the Yangtze River, and as shown from Figure 13f–j, the ferry is very close to the shore, of which the speed is very fast, and the image resolution is also 640 × 360 px. It can be seen from the figures that since ferry sailing has a great influence on the water surface, the line segments have all been removed from the line segment pool in the tenth frame, and the rough selection has achieved good results.

**Figure 14:**
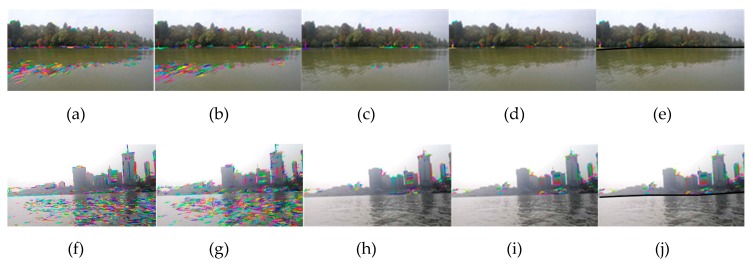
The WSL generation experiments for large scenes: (**a**–**e**) is experiment III in the East Lake, which is line segment extraction, line segment matching, line segment pool rough selection, line segment pool fine selection and the WSL generation. (**f–j**) is experiment IV, which is in the Yangtze River.

Experiments III and IV are similar to experiments I and II; as shown in Figure 14, the first line is in the East Lake waters, and the second line is in the Yangtze River waters. Compared with experiment I and II, ships are farther away from the shore, shoreline, and buildings become blurred in the image. The resolution of the image is 1280 × 720, selected by the energy function. It can be seen from the figures that the WSL can also be accurately detected for large scenes.

**Figure 15:**
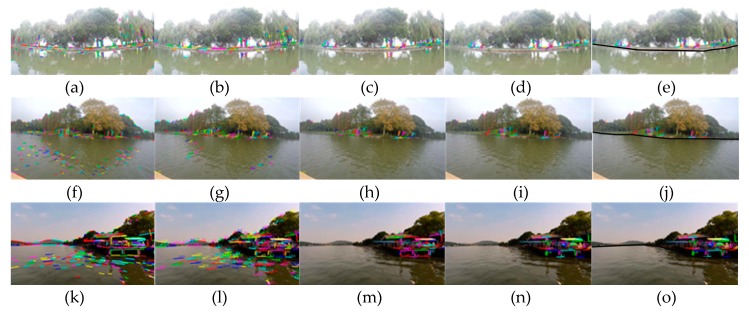

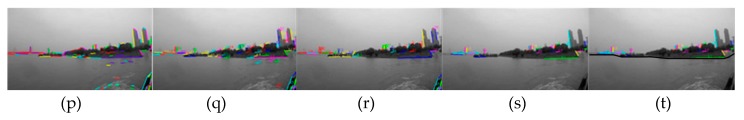
The second group of the WSL detection experiments in the case of non-straight line: (**a**–**e**) and (**f**–**j**) are experiments in sunny days, the five columns from left to right correspond to line segment extraction, line segment matching, line segment pool rough selection, line segment pool fine selection and WSL generation. (**k**–**o**) and (**p**–**t**) are experiments in cloudy days.

The second group of experiments is shown in Figure 15. The first and second rows correspond to sunny days, and the third and fourth rows correspond to cloudy days. The five columns from left to right correspond to line segment extraction, line segment matching, line segment pool rough selection, line segment pool fine selection and WSL generation.

It can be seen from the Figure 15 that the water surface is relatively calm in the East Lake. In the tenth frame (Figure 15c), there are five remaining line segments in the water surface, which can be removed by fine selection, while the other images can achieve good results only by rough selection. From the experimental results, we can see that whether the WSL is curved caused by camera lens distortion (the first line), or because the WSL itself is a non-straight line (the second and third lines), and for the partial occlusion in the camera field of vision (the fourth line), the accurate onshore line segment pool can be obtained by the coarse-to-fine strategy. In addition, this method does not use straight line fitting, so it can complete detection well, whether the WSL is straight or not.

### 4.2. Error Analysis

**Figure 16:**
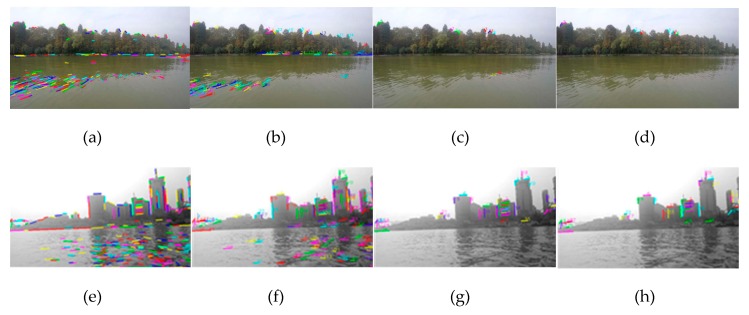
The experimental results of low-resolution images (640 × 360 px): (**a**–**d**) is experiment corresponding to the first group experiment III, which is line segment extraction, line segment matching, line segment pool rough selection and line segment pool fine selection. (**e**–**h**) is experiment corresponding to the first group experiment IV.

Firstly, in order to verify the significance of selecting image resolution through energy function, experiments are carried out with low-resolution images for large scenes. For example, in the first group of experiments, the resolution of 640 × 360 px is used, instead of 1280 × 720 px, for experiments III and IV, and the results of the experiments are obtained as shown in Figure 16. Compared with Figure 14d and Figure 16d, it is obvious that the former is better. Although the latter can remove the line segments in the water area very well, it also greatly reduces many line segments in the non-water area. It is not difficult to imagine that the obtained WSL will have a large deviation.

**Figure 17:**
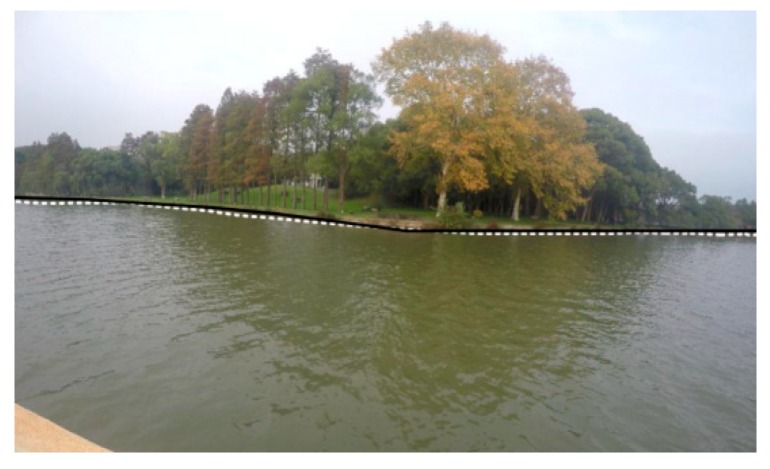
Comparisons between the WSL obtained by the method and the true value; the black line is the result of the method, and the white dot line is the true value obtained by hand.

Then, the numerical method is adopted to calculate the accuracy of this method. The detection results of Figure 15j are compared with the true data, which are obtained by hand in the original image according to the pixels. As shown in Figure 17, the black line is the result of the proposed method, and the white dot line is the true value. It can be seen that the detected WSL is very close to the true value. We adopt the method of reference [8] to calculate evaluation errors by Equations (7) and (8). The average difference is 2.1 pixels, the maximum difference is seven pixels, and the standard deviation is 5.4 pixels. The computational equation is as follows:

(7) $$E_{1}=\frac{1}{n}\sum_{k=1}^{n}\left(H_{k}-{\tilde{H}}_{k}\right),$$

(8) $$E_{2}=\sqrt{\frac{1}{n}\sum_{k=1}^{n}{\left(H_{k}-{\tilde{H}}_{k}\right)}^{2}},$$

where n is the sampling point of the WSL, $H_{k}$ is the k-point ordinate of the true value, and ${\tilde{H}}_{k}$ is the *k*-point ordinate of the method.

**Figure 18:**
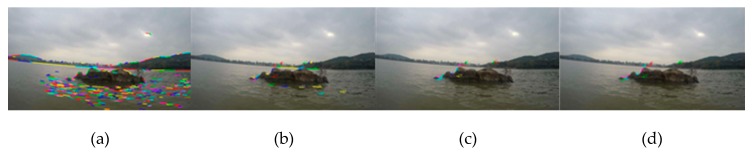
The failure case of this method: (**a**–**d**) are respectively line segment extraction, line segment matching, line segment pool rough selection and line segment pool fine selection.

Of course, there are also failures. As shown in Figure 18, if there are interferences in the close range of large scene, which cannot be removed by the rough selection of water area instability and the fine selection of epipolar constraint between image frames, then most of the line segments in the pool will be concentrated on the interferences. Figure 18a–d correspond to line segment extraction, line segment matching, rough selection and fine selection, respectively. As can be seen from the figures, the proposed method in this paper cannot remove these interferences, so it is also impossible to get the desired WSL.

### 4.3. Parameter Value Analysis

**Figure 19:**
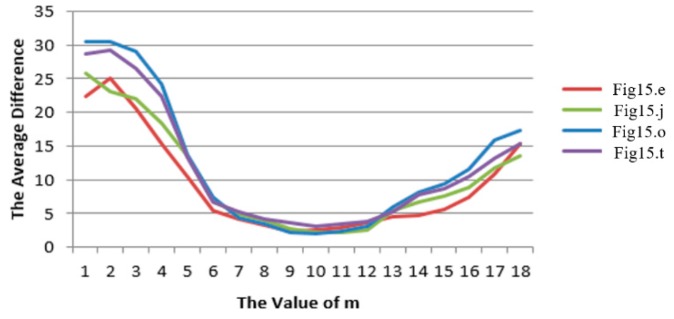
Parameter value analysis: the relationship between m value and average difference.

There are two main control parameters, such as $t_{i-m}$ and${\;t}_{i+n}$. Because these two time periods have equal contributions to the construction of the line segment pool, it is worthwhile to make them equal. By changing the parameter, the average difference of the method performed on the first column of Figure 15 are as shown in Figure 19. Setting a smaller m results in a larger amount of retained interference, which not only increases the time for the fine selection operation, but also increases the average difference. Setting a larger m resulted in fewer line segments to be retained, which in turn affects the accuracy of the algorithm, and also increases overall operation time, because capturing just one frame takes 33 ms, let alone the rest. Both of these caused decreased detection accuracy.

By comparing Figure 5, Figure 6 and Figure 19, it is found that the optimal m value appears at the lifetime of line features in water area, so for the convenience of engineering, the values of m and n are uniformly 10.

### 4.4. Comparison with Other Methods

**Figure 20:**
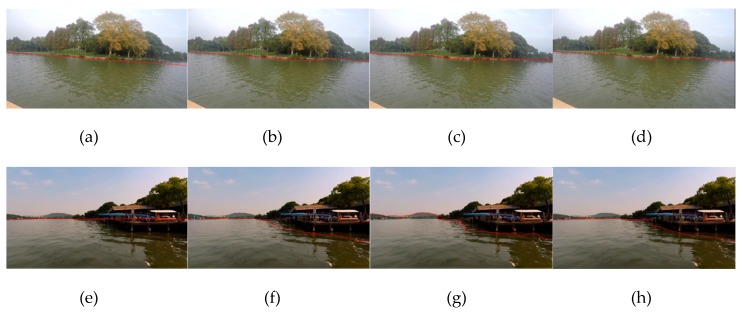
The WSL detection results of four different algorithms: (**a**–**d**) are experimental results with good illumination, respectively corresponding to straight line fitting (SLF) [10] method, edge detection combined with the threshold segmentation (EDS) [9] method, superpixel segmentation (SPS) [14] method and online learning (OL) [10] method. (**e**–**h**) are experimental results with bad illumination.

The performance of the different algorithms is compared via Figure 15f,k, whose WSLs are somewhat curved. As shown in Figure 20, the four columns correspond to different methods respectively, which are respectively SLF [10] method, EDS [9] method, SPS [14] method and OL [17] method. The first column uses straight line fitting combined with the RANSAC method (SLF) [10]. Obviously, the SLF method is not suitable, and the obtained WSL is far from the true value. The second column uses edge detection combined with the threshold segmentation method (EDS), and edge detection method used is the canny algorithm [9]. The WSL in Figure 20b is nice, due to good illumination. However, when the illumination condition is so bad that both the buildings on the bank and their shadows are dark, it is difficult to judge where the WSL lies. As shown in Figure 20c, the detected WSL is quite different from the true value. Similarly, for images with bad illumination, the superpixel segmentation method (SPS) [14] cannot distinguish whether the shadow is shore or water area, so what is obtained in Figure 20g is not a satisfactory result.

The results obtained by online learning method (OL) [17] are shown in Figure 20d,h. The method first uses lidar and vision to judge the input image pixels, and then the image pixels are fed into a convolutional neural network (CNN) to train the network online, and finally, the online trained CNN is used to segment the input image. The OL method has a good performance, but it is too time-consuming.

The above are qualitative comparisons; in order to quantitatively evaluate the performance of the proposed method, various these detection methods are adopted in the Figure 15k to compare the respective accuracy and running time, and the average difference and standard deviation are used to measure the accuracy performance of the method. As observed in Table 4, the accuracy of this method is superior to that of the other four methods. The running time of various methods is also calculated, and this method takes longer than other methods, except for the OL method. Due to the fact that the method deals with sequence images with a time span of 10 frames, and the calculation of line segment matching is large, the method has no advantage in time consumption, but its significance is to make use of the essential characteristics of water area in sequence images.

## 5. Conclusions

In this paper, a novel three-step approach for WSL detection based on image sequence is proposed and validated by field experiments in inland river scenes. Firstly, the initial line segment pool is built by the LSD algorithm. Next, the onshore line segment pool is obtained by the coarse-to-fine strategy, in which the line segment pool is roughly selected by the instability of the water area, and the stable line segment pool is finely selected by the epipolar constraint between the sequence images. Then, the onshore line segment pool of the multi-frame image generates the shore area, and the lower boundary is the desired WSL. In the end, field experiments and results illustrated the performance of the WSL detection method. Compared with other methods, the method is more adaptable, and can detect both the straight WSL and the curved WSL. 

Therefore, our method can potentially be used to ensure the navigation safety of the USV. However, there is still a lot of room for improvement in the future, for example, WSL detection and navigable area detection are combined for USV autonomous navigation, which is the next research content.

## Figures and Tables

**Table 1:** Experimental equipment.

Equipment	Parameter	Value/Model
USV	Length	4.15 m
	Width	1.60 m
	Deflection angle	−25°~25°
	Max speed	2.2 m/s
Computer	CPU	Intel i5-6600
	Memory	8 GB

**Table 2:** Experimental sites and data.

Site	Water Speed	Ship Speed	Ship	Weather	Data Set
Site 1	0–0.3 m/s	0–2 m/s	USV	Sunny	data 1
				Cloudy	data 2
				Rainy	data 3
Site 2	1–1.5 m/s	0–5 m/s	ferry	Sunny	data 4
				Cloudy	data 5
				Rainy	data 6
Site 3	1–1.5 m/s	0–5 m/s	ferry	Sunny	data 7
				Cloudy	data 8
				Rainy	data 9

**Table 3:** Experimental conditions.

Exp. ID	Riverbank Distance	Sites	Speed
I	Close (<50 m)	The East Lake	Slow (<2 m/s)
II	Close	The Yangtze River	Fast (≥2 m/s)
III	Far (≥50 m)	The East Lake	Slow
IV	Far	The Yangtze River	Fast

**Table 4:** WSL detection performance comparisons.

Algorithm	Average Difference (pixels)	Standard Deviation (pixels)	Running Time (ms)
SLF [10]	11.2	26.4	232
EDS [9]	6.5	14	194
SPS [14]	8.3	20.5	151
OL [17]	4.6	8.1	5436
This Method	2.7	5.8	2647
